# Oxidative Stress in Chronic Hepatitis B—An Update

**DOI:** 10.3390/microorganisms10071265

**Published:** 2022-06-21

**Authors:** Gabriela Loredana Popa, Mircea Ioan Popa

**Affiliations:** 1 Department of Microbiology, “Carol Davila” University of Medicine and Pharmacy, 020021 Bucharest, Romania; mircea.ioan.popa@gmail.com; 2 “Cantacuzino” National Military Medical Institute for Research and Development, 011233 Bucharest, Romania

**Keywords:** oxidative stress, hepatitis B virus infection, hepatocellular carcinoma, fibrosis

## Abstract

In recent years, the role of oxidative stress has been investigated in an increasing number of infections. There is a close link between the inflammation that accompanies infections and oxidative stress. Excessive reactive oxygen species induce harmful effects on cell components, including lipids, proteins, and nucleic acids. A growing body of evidence attests to the role of oxidative stress in the pathogenesis of viral liver infections, especially in hepatitis C virus (HCV) infection. Regarding hepatitis B virus (HBV) infection, the data are limited, but important progress has been achieved in recent years. This review presents the latest advances pertaining to the role of the oxidative stress byproducts in the pathogenesis of chronic hepatitis B, constituting a source of potential new markers for the evaluation and monitoring of patients with chronic hepatitis B.

## 1. Introduction

Oxidative stress represents the imbalance between reactive oxygen species (ROS) and antioxidant systems [1]. Redox balance is essential to maintain cell homeostasis. Mitochondria are the main endogenous source of ROS—the free radicals being generated during mitochondrial respiration—in the cell [2,3]. Other endogenous sources are the endoplasmic reticulum (ER) and peroxisomes [4,5]. There are also many exogenous sources, including cigarette smoke, pesticides, radiation, certain foods, etc. [4]. Free radicals are molecules that have unpaired electrons in their structure, which give them high reactivity [6]. At low/moderate concentrations, ROS exert beneficial effects in the human body by participating in numerous signaling pathways [6,7]. At high concentrations, they have harmful effects on cell components (e.g., lipids, proteins, nucleic acids, and carbohydrates). These compounds lose their functionalities due to oxidation processes, and can modify the physiological redox state, which influences H_2_O_2_ signaling in various cell processes, such as proliferation and differentiation [8,9]. Therefore, lipid peroxidation generates reactive aldehydes such as 4-hydroxy-2-nonenal (4-HNE), malondialdehyde (MDA), and isoprostanes. The main byproducts of glycoxidation are the advanced glycation end products (AGEs), and protein carbonylation is a major hallmark of protein oxidation. DNA oxidation leads to several mutagenic lesions, the most common being 8-hydroxydeoxyguanosine (8-OHdG) [10,11,12,13]. The harmful effects of oxidative stress can be counteracted by antioxidant molecules. Antioxidant systems include endogenous antioxidants (e.g., superoxide dismutase (SOD), catalase (CAT), glutathione peroxidase (GPX), peroxiredoxins) and exogenous antioxidants (vitamin C, tocopherols, etc.) [6,7]. Endogenous antioxidants are classified into two main groups: enzymatic (SOD, CAT, GPX, etc.) and non-enzymatic (glutathione, L-arginine, bilirubin, transferrin, coenzyme Q10, melatonin, etc.) [14]. However, in some instances, the antioxidant capacity is exceeded, which allows the perpetuation of oxidative stress and the initiation of pathological processes, such as chronic inflammation or carcinogenesis. Today, it is well known that oxidative stress is involved in the pathogenesis of many diseases, and modern therapies try to limit the oxidative damage [15,16,17]. There are many studies that emphasize the involvement of oxidative stress in hepatitis C virus (HCV) infection, but in hepatitis B the data are less numerous [18,19,20]. However, in recent years, there has been growing evidence of the role of oxidative stress in hepatitis B virus (HBV) infection.

HBV infection is still a substantial public health problem, although it can be prevented by vaccination, and effective viral-suppressive medications are available. The main route of transmission is through direct contact with infected blood or body fluids [21]. HBV is an enveloped, partially double-stranded DNA virus that is classified in the *Hepadnaviridae* family. Ten genotypes of HBV (A to J) have been identified, with different geographical distribution and distinct characteristics of the disease in terms of chronicity rates, clinical outcomes, and responses to therapy [22]. Clinical manifestations of HBV infection include acute hepatitis, chronic hepatitis, liver cirrhosis, and hepatocellular carcinoma. The evolution of the infection mainly depends on the interaction between the virus and the host’s immune system [23,24]. Some individuals have inactive hepatitis B that does not require therapy, while in other patients the disease progresses to liver cirrhosis and hepatocellular carcinoma [23,25]. When infection occurs early in life, it is associated with a higher rate of chronicity; therefore, in the case of infants, the progression to a chronic form occurs in 90% of cases, while in adults the percentage varies between 5 and 10% [26]. In 2019, 29,996 cases of HBV infection were reported in 30 EU/EEA member states, of which 48% were classified as cases of chronic hepatitis. These data show that prevention programs need to be improved to achieve the goal of eliminating hepatitis B [27]. In subjects who develop chronic hepatitis, the function of the cells responsible for the antiviral defense—such as Kupffer cells, dendritic cells, or natural killer cells—is altered. In such cases, the ability of these cells to produce a series of cytokines involved in antiviral defense is diminished, resulting in a tolerogenic liver microenvironment. The activity of CD4+ and CD8+ cells is also impaired [23].

The occurrence of gene mutations is known to be one of the main mechanisms that allow HBV to survive and escape the host’s immune response. It seems that oxidative stress could contribute to the development of mutations responsible for resistance to therapy. Elevated oxidant levels and deficient antioxidant defense are correlated with HBV gene mutations [28]. Furthermore, the degree of oxidative stress damage may be correlated with the HBV genotype. Genotype C has been associated with more pronounced oxidative stress [28,29].

HBV interacts with antioxidant defense mechanisms. It induces the activation of the Nrf2/ARE pathway of antioxidant defense, but also modulates the activity of enzymes independent of the Nrf2/ARE pathway, such as the glutathione-S-transferase isoforms, peroxiredoxin 1, and SOD2. On the other hand, HBV inhibits the expression of some proteins involved in antioxidant defense, such as selenoprotein P and selenium-binding protein 2 [30]. The oxidant–antioxidant balance seems to play a significant role in modulating HBV replication and infectivity. According to a recent study, H_2_O_2_ induces viral replication, while N-acetyl-cysteine—a compound with antioxidant properties—decreases viral replication [31]. Ren et al. showed that sirtuin 3—an important mitochondrial deacetylase—decreases the levels of HBx-induced ROS in the cell and, thus, inhibits viral replication [31]. The study by Kim et al. indicated that ROS promote capsid formation in the presence of the Hsp90 complex. In addition, the study revealed that GSH inhibits Hsp90-driven HBV capsid assembly [32]. The aim of this review is to summarize current knowledge on the involvement of oxidative stress in HBV infection, and to present the latest studies (2017–2021) that have analyzed oxidative stress markers in patients with chronic hepatitis B to provide new insights into the role of oxidant–antioxidant balance in the pathogenesis of HBV infection.

## 2. Liver and Oxidative Stress

The liver is a major organ subjected to oxidative stress. Oxidative stress is involved in the pathogenesis of inflammatory, metabolic, and proliferative liver diseases. Chronic liver disease is usually associated with high levels of oxidative stress, regardless of its etiology [33,34,35]. The parenchymal cells are the initial cells that are exposed to the harmful effects of oxidative stress. In addition, Kupffer cells, hepatic stellate cells (HSCs), and endothelial cells are very sensitive to oxidative stress [36]. In the liver, hepatocytes represent an important source of ROS that are generated in the mitochondria and endoplasmic reticulum through several reactions catalyzed by cytochrome P450 enzymes [33,37]. Oxidative stress induces the production of pro-inflammatory cytokines in Kupffer cells, contributing to the development of an inflammatory process [36]. The activated Kupffer cells stimulate NF-κB in hepatocytes, which promotes an increase in IL-6 levels and STAT-3 activation [38]. Furthermore, ROS induce the release of profibrogenic cytokines that can activate HSCs. Under oxidative stress conditions, the proliferation of HSCs and collagen synthesis are increased, favoring the development of fibrosis [36]. A recent work highlighted the role of cytoglobin in the catabolism of H_2_O_2_ and lipoperoxides in HSCs, which are compounds that induce their activation [37]. Lipid peroxidation in HSCs has been shown to stimulate pro-collagen-type-I synthesis [36,39]. Increased lipid peroxidation plays a major role in liver damage, leading to inflammation, abnormal hepatocyte permeability, and even liver cytolysis [40]. Additionally, high levels of ROS alter the permeability of the mitochondrial membrane, which allows the release of pro-apoptotic molecules [33]. Necrotic hepatocytes have been shown to release mitochondrial DNA (mtDNA), which promotes an inflammatory process via TLR9 and cGAS-STING. Activation of cGAS-STING results in the release of IFN 1 from inflammatory cells, which can trigger oxidative stress [41]. ROS can accumulate in a cell where mtDNA depletion has occurred [42].

Liver cancer may be considered to be an inflammation-induced malignancy. The development of liver cancer involves chronic liver inflammation in the context of a liver disease such as viral hepatitis B, viral hepatitis C, metabolic disorders, excessive alcohol consumption, etc. The close link between chronic inflammation and oxidative stress is well known. Oxidative stress is a key event in hepatocellular carcinogenesis. Therefore, highly reactive molecules react with DNA bases, resulting in pro-mutagenic DNA adducts. Oxidative stress has been shown to contribute to cell migration, invasion, and metastasis in hepatocellular carcinoma [43,44,45].

## 3. The Link between HBV Proteins and Oxidative Stress

The HBV genome contains four partially overlapping open reading frames (ORFs) that encode viral polymerase, surface antigen, core, and X proteins [22,46]. The main viral proteins that can manipulate the balance between oxidants and antioxidants are HBx, HBsAg, and HBeAg [30,47].

### 3.1. HBx Protein

HBx is a 154-amino-acid protein that is essential for HBV replication, and has been reported to be associated with the covalently closed circular DNA (cccDNA). HBx regulates many processes, including gene transcription, epigenetic modifications, signal transduction, apoptosis, etc. [48,49]. A recent study on transgenic HBx mice revealed that HBx is a multifaceted molecule that is involved in hepatic steatosis, hepatic fibrosis, and disorders of the carbohydrate metabolism [50]. HBx may cause an increase in the level of oxidative stress through mitochondrial dysfunction. The protein acts on mitochondrial complex 1 and alters the electron transfer, resulting in the accumulation of quinone species that interact with the oxygen molecules generating ROS [51]. HBx promotes oxidative stress by activating calcium-dependent signaling and cell kinases, resulting in the upregulation of NF-κB and STAT-3 [52]. Using a murine model expressing HBx, Ling et al. revealed that HBx increases the expression of IL-6, IL-1β, and IL-18, which contribute to the formation of an inflammatory microenvironment in the liver. The pro-inflammatory cytokines drive the production of ROS, which further augment the inflammatory process, creating a vicious circle that results in liver injury [53]. However, it should be noted that in the early stages of infection, HBV acts as a stealth virus, and does not induce any genes during entry and replication into the host cells. The virus spreads before the activation of the adaptive immune response, which is initiated a few weeks later. Moreover, HBV-specific T-cell response is low in chronic hepatitis B [54].

Furthermore, HBV-induced ROS production triggers Snail-mediated epigenetic silencing which, in turn, leads to the suppression of cytokine signaling suppressor 3 (SOCS3)—a process connected with the activation of IL6/STAT-3 (a pathway that plays a role in carcinogenesis) [55]. Under oxidative stress conditions, Raf-1 is translocated to the mitochondria. Raf serine/threonine kinases exist as three isoforms: A-Raf, B-Raf, and C-Raf (also known as Raf-1). In the mitochondria, Raf-1 is involved in protecting cells from stress-mediated apoptosis. HBx is capable of forming a protein–protein complex with Raf-1, and inhibits the apoptosis of infected hepatocytes [52]. There are several studies on the role of HBx in apoptosis, but the results are confusing [56,57]. HBx has been shown to induce apoptosis, but also to inhibit it. It has been hypothesized that HBx needs cofactors such as tumor necrosis factor (TNF)-α, Fas, or oxidative stress to induce apoptosis [58]. Recently, Gao et al. have revealed that HBx enhances the susceptibility of normal hepatocytes to oxidative-stress-induced apoptosis by modulating the mitochondrial permeability transition pore (MPTP). They showed that apoptosis is induced by the translocation of Bax—a pro-apoptotic protein of the Bcl2 family [58]. Ma et al. found that translocation of Bax to the mitochondria occurs through its interaction with the voltage-dependent anion channel (VDAC)-2. VDAC, also known as a mitochondrial porin, is considered to be part of the MPTP [59]. The HBx protein also induces ER stress. The activation of the IRE1-XBP1 and ATF6 UPR pathways is related to HBx-mediated stress in the ER [60]. Furthermore, HBx alters antioxidant defense through several mechanisms. It modifies the structure of NQO1—a protein with antioxidant effects—through the methylation of its promoter. HBx has also been shown to lower glutathione levels in hepatoma cells. In this context, the Nrf2/ARE pathway is activated to restore the balance between oxidants and antioxidants [61].

### 3.2. HBe Protein

Hepatitis B core-related antigen (HBcrAg) is composed of three proteins (hepatitis core antigen (HBcAg), hepatitis e antigen (HBeAg), and a small core-related protein (p22cr)) coded by the precore/core region [62]. Data on the relationship between HBe and oxidative stress generation are scarce and inconclusive. In vitro, it has been shown that HBeAg downregulates LPS-induced NLRP3 inflammasome activation and IL-1β production in Kupffer cells. This process is achieved on the one hand by inhibiting NF-κB, which leads to the suppression of the NLRP3 inflammasome signaling pathway and pro-IL-1β expression; on the other hand, caspase-1 activation and IL-1β maturation are blocked through the inhibition of ROS generation. The NLRP3 inflammasome plays a pivotal role in antiviral defense [47]. Data from an in vivo study have shown significantly lower levels of the antioxidants SOD and vitamin C in HBeAg-positive patients than in patients with inactive disease. Further studies should be performed to better understand the link between the HBe protein and oxidative stress [63].

### 3.3. HBs Protein

HBsAg includes three proteins: a small protein (sHBsAg), a middle protein (mHBsAg), and a large protein (lHBsAg). The pre-S1 mRNA and the pre-S2/S mRNA encode the three proteins of HBsAg, which form the viral envelope [62,64]. PreS1 and preS2 deletions in ground-glass hepatocytes are associated with abnormal retention of mutant large and middle surface proteins in the ER [65]. Moreover, some naturally occurring mutants of the small HBsAg show a reduced ability to be secreted and accumulate in the ER [30]. Abnormal retention of mutants in the ER may promote stress in this organelle [60]. Under conditions of oxidative stress, in the ER, the unfolded protein response is activated, and COX-2 expression is increased, which exerts a pro-inflammatory effect [50]. In Huh7 cells harboring preS mutations, elevated levels of ROS have been identified, and have been associated with oxidative DNA damage, which can enhance genomic instability and contribute to the development of a malignant process [60].

## 4. New Advances in Understanding the Role of Oxidative Stress in HBV Infection

In 2016, Alavian et al. performed a review that included 18 studies evaluating lipid peroxidation in patients with HBV infection. Most of the studies assessed the serum levels of MDA as a marker of lipid peroxidation, and identified significantly higher levels compared to healthy subjects. In the same review, the studies available on protein and DNA oxidation were analyzed; on this topic, data are scarce, but indicate that excessive levels of ROS exert negative effects on these components in patients with HBV infection [66]. These results were also confirmed in the study conducted by Pomacu et al. in 2021, which revealed high serum levels of thiobarbituric-acid-reactive substances (TBARSs)—a lipid peroxidation marker—and high serum levels of carbonylated proteins resulting from protein oxidation, in patients with liver cirrhosis related to HBV and HCV infection. Regarding the level of the total antioxidant capacity (TAC), there were no significant differences between patients with liver cirrhosis and the control group. The authors explain this result by the fact that viral components stimulate the activity of antioxidant enzymes such as catalase and glutathione peroxidase, while on SOD isoenzymes they exert an inhibitory effect, and TAC is a cumulative marker of oxidative stress [67]. Huang et al. emphasized the role of copper (Cu) homeostasis in the pathogenesis of HBV infection. They studied Cu homeostasis in patients with chronic hepatitis B, and identified elevated serum levels of total Cu and increased amounts of urinary Cu, considering that this may have been an adaptive mechanism in those patients. However, serum Cu ion (Cu^+^) levels were lower in patients with chronic hepatitis B compared to healthy subjects, which may have been the result of oxidative stress conditions that led to the conversion of Cu^+^ to Cu^2+^. The levels of Cu-containing enzymes, ceruloplasmin, and SOD were elevated, but their activity was reduced, suggesting poor antioxidant defense in those patients. Cu can be dissociated from ceruloplasmin under oxidative stress conditions, leading to decreased activity of the enzyme [68].

Genetic factors might contribute to the susceptibility to oxidative stress. Ma et al. studied the polymorphism of certain genes that modulate the activity of enzymes responsible for the oxidation and reduction processes, along with their main effects on the hepatitis B virus. The results of the study, which included 3128 Han Chinese individuals divided into five groups—healthy subjects, patients with chronic hepatitis B, patients with liver cirrhosis, patients with hepatocellular carcinoma, and patients with natural clearance—showed that cytochrome B-245 alpha chain (CYBA)-rs4673AG and glutamate–cysteine ligase modifier subunit (GCLM)-rs41303970A were associated with HBV-induced liver disease, while neutrophil cytosolic factor 4 (NCF4)-rs1883112G allele and NADPH oxidase 4 (Nox4)-rs1836882 TC were encountered more frequently in healthy controls [69].

In recent years, researchers have focused on detecting new markers for assessing oxidative stress in HBV infection (Table 1). Wang et al. were the first to demonstrate the methylation of the type I interferon receptor (IFNAR) in chronic hepatitis B, and showed that this process is influenced by oxidative stress. The study revealed higher levels of MDA in the methylated group with chronic hepatitis B than in the unmethylated group, while the glutathione levels were lower in the methylated group compared to the unmethylated group. Oxidative stress can exert a destructive effect on protein structure and lead to decreased IFNAR levels, resulting in upregulation of IFNAR gene expression. Methylation under oxidative stress has been identified in several cancers, including hepatocellular carcinoma [70].

Xiong et al. proposed NADPH oxidase-2 (NOX-2) as a new marker of HBV-related disease. NOX-2 is considered to be an important modulator of ROS production. The study included patients with disorders caused by HBV infection (i.e., chronic hepatitis, cirrhosis, and hepatocellular carcinoma) and healthy subjects, and serum NOX-2 levels were significantly higher among patients. A positive correlation was also observed between NOX-2 and SOD, interferon-stimulated IL-6 gene 15, alkaline phosphatase, and gamma-glutamyl transpeptidase (GGT) [71]. Recently, Yang et al. detected higher serum levels of soluble E-cadherin (sE-cadherin) in patients with chronic hepatitis B compared to healthy subjects. Additionally, sE-cadherin was positively correlated with oxidizing compounds (e.g., MDA, TOS, NOX-2) and negatively correlated with antioxidant systems (e.g., TAC, SOD, glutathione-GSH). The authors proposed sE-cadherin as a marker of oxidative stress in HBV [72]. The levels of sE-cadherin have been shown to be higher in patients infected with HBV compared to controls. In patients with chronic hepatitis B and cirrhosis, the levels of sE-cadherin were positively correlated with IFN-γ and transaminases [73].

Murad et al. revealed that blood levels of glutamine and nitrotyrosine were significantly higher in untreated and treated patients with chronic hepatitis B compared to a control group, but with no significant differences between treated and untreated patients, and suggested that further studies would be needed to establish the roles of the two markers in predicting the response to therapy [74]. Glutamine is a precursor of GSH, and exhibits antioxidant and anti-inflammatory properties. Elevated glutamine levels are associated with increased amounts of ammonia, which may contribute to the development of hepatic encephalopathy. Glutamine is transported to the mitochondrial level, where it is transformed into ammonia—a process that leads to the generation of oxidative stress by altering the permeability of the inner mitochondrial membrane [74,75]. Nitrotyrosine is a marker of oxidative stress, and is generated by peroxynitrite-mediated nitration of tyrosine residues [74].

In the last decade, thiol–disulfide homeostasis has been studied in many disorders, and it is considered a novel marker of oxidative stress. Celik et al. assessed thiol–disulfide homeostasis in patients with chronic hepatitis B, and found lower serum thiol levels compared to the control group. With regard to serum disulfide levels, no significant differences were identified. The authors found a negative correlation between thiol levels and total anti-HBc IgG, and a positive correlation between DS/NT and DS/TT ratios and total anti-HBc IgG [76].

**Table 1:** Potential markers of oxidative stress in chronic hepatitis B (studies published between 2017 and 2021).

Markers	Groups	Reference
Total Cu, Cu ions, small-molecule Cu, ceruloplasmin, SOD-1, urinary Cu	32 patients with chronic hepatitis B; 10 healthy subjects	Huang et al. (2018) [68]
NOX-2	105 patients with chronic hepatitis B; 58 patients with HBV-related cirrhosis; 48 patients with HBV-related hepatocellular carcinoma; 104 healthy subjects	Xiong et al. (2018) [71]
sE-cadherin, TAC, GSH, SOD, TOC, NOX-2, MDA	51 patients with HBeAg-negative chronic hepatitis B; 54 patients with HBeAg-positive chronic hepatitis B; 109 healthy individuals	Yang et al. (2020) [72]
NT, TT, DS, DS/NT. DS/TT, NT/TT	63 patients with chronic hepatitis B; 60 healthy subjects	Celik et al. (2020) [76]
Glutamine, nitrotyrosine	50 patients with untreated chronic hepatitis B; 50 patients with untreated chronic hepatitis C; 50 patients with treated chronic hepatitis B; 50 patients with treated chronic hepatitis C; 50 healthy subjects	Murad et al. (2021) [74]
MDA, 4-HNE, carbonylated proteins, TAC	25 patients with alcoholic cirrhosis; 10 patients with HBV and HCV related cirrhosis; 10 healthy subjects	Pomacu et al. (2021) [67]

Cu: copper, SOD: superoxide dismutase, NOX: NADPH oxidase, sE-cadherin: soluble E-cadherin, TAC: total antioxidant capacity, GSH: glutathione, TOC: total oxidant capacity, MDA: malondialdehyde, 4-HNE: 4-hydroxynonenal, NT: native thiol, TT: total thiol, DS: disulfide, HBV: hepatitis B virus, HCV: hepatitis C virus.

## 5. Oxidative Stress—A Potential Source of New Markers for Hepatic Fibrosis Assessment

The main mechanism by which fibrosis occurs is based on the production of an increased amount of extracellular matrix by HSCs, which accumulates in the liver parenchyma and alters its homeostasis [77]. Staging of fibrosis is an important step in the management of patients with HBV infection; it is used to establish the timing of the initiation of therapy, and to predict the long-term evolution of the disease. Currently, for the evaluation of fibrosis, the gold standard method is liver biopsy—an expensive and invasive maneuver; therefore, the implementation of new noninvasive markers is necessary [78,79]. The aspartate transaminase (AST)-to-platelet ratio index (APRI) and the fibrosis index based on four factors (FIB-4) are the most widely used serum-based markers to assess the degree of liver fibrosis [78,80]. Other serum tests to describe the degree of fibrosis are BARD, GPR, ELF, etc. [81]. However, these markers have a number of disadvantages, such as low sensitivity and difficult calculation, and their results can be influenced by various factors (e.g., the administration of drugs that influence the levels of transaminases) [82,83]. Among imaging-based methods for the evaluation of fibrosis, transient elastography is widely employed, but it is difficult to use in obese patients or in those with ascites [83]. According to a recent study, magnetic resonance elastography is a better method to evaluate fibrosis than two-dimensional shear-wave elastography [78]. In this context, the researchers focused on identifying new markers that would be useful for assessing fibrosis in patients with chronic hepatitis B (Table 2).

Duygu et al. suggested that the levels of oxidative stress could be correlated with hepatitis B activity. They showed that the serum levels of oxidative stress markers, TOC, lipid hydroperoxides, and oxidative stress index (OSI) were higher, while the levels of antioxidant compounds, CAT, ceruloplasmin, and TAC were lower, in patients with chronic hepatitis B compared to patients with an inactive HBsAg carrier state. There was no association between the OSI or histological activity index and fibrosis levels. The authors explained that this was due to the homogeneity of the patients in whom the biopsies were performed, with the patients with liver cirrhosis being excluded from the study; all patients presented moderate chronic active hepatitis [84]. However, Wang et al. evaluated the relationship between oxidant–antioxidant balance and the extent of hepatic fibrosis, and showed a negative correlation between the TAC level and the degree of fibrosis. Furthermore, the TAC sensitivity was higher compared to APRI (73.91% vs. 56.52%). The authors found that TAC could be a marker of fibrosis in inactive carriers and APRI in the active carriers [85]. For the evaluation of liver fibrosis, another promising marker is urinary 8-oxo-7,8-dihydroguanosine (8-oxo-Gsn). Higher levels of 8-oxo-Gsn and 8-oxo-dGsn, resulting from the oxidative damage to RNA and DNA, respectively, were detected in urine from HBV-infected patients compared to the control group. The study found that patients with elevated urinary levels of 8-oxo-Gsn have a higher risk of having advanced liver fibrosis. An association was also found between urinary 8-oxo-Gsn and the following parameters: APRI, AST, and PT [86].

Dertli et al. analyzed patients with chronic hepatitis B, patients with liver cirrhosis associated with hepatitis B, and healthy subjects, and showed a progressive decrease in the serum levels of thiols (total and native thiols) in correlation with liver fibrosis stage. Regarding serum disulfide levels, significantly higher levels were detected in patients with chronic hepatitis B compared to the control group, but the differences were insignificant between the groups. The authors suggested that this was due to low albumin levels, with thiol albumin accounting for a significant proportion of the plasma thiol pool [87].

**Table 2:** New promising markers of oxidative stress for assessing liver fibrosis.

Markers	Groups	Reference
8-oxo-Gsn	138 patients with HBV infection; 169 healthy subjects	Xu et al. (2018) [86]
TT, NT, DS, DS/NT, DS/TT, NT/TT	71 patients with chronic hepatitis B; 50 patients with HBV-related cirrhosis; 45 healthy subjects	Dertli et al. (2018) [87]
TAC	54 patients with HBV-related cirrhosis	Wang et al. (2021) [85]

8-oxo-Gsn: 8-oxo-7,8-dihydroguanosine, TAC: total antioxidant capacity, NT: native thiol, TT: total thiol, DS: disulfide, HBV: hepatitis B virus.

## 6. Oxidative Stress—A Cofactor in HBV-Related Carcinogenesis

The HBV genome integrates within the coding sequence or close to an array of key regulatory cellular genes that can deregulate proto-oncogenes and tumor-suppressor genes. High ROS concentrations influence the expression of these genes, and can lead to mutations in the host cell genome, as well as to alterations in various signaling pathways and, eventually, to a malignant phenotype [88]. Chronic inflammation related to HBV infection is characterized by high levels of pro-inflammatory cytokines such as IL-1β, IL-6, CXCL-8, and TNF-α that promote an oxidative environment [45]. The C-terminal region of HBx induces the generation of ROS, resulting in mitochondrial DNA injury—an event that may underlie the development of hepatocellular carcinoma [42]. The role of oxidative stress in liver cancer has been highlighted in several studies on hemochromatosis [89,90]. Lipid peroxidation results in byproducts such as MDA and HNE, which form pre-mutagenic DNA adducts with DNA bases. In addition, HNE adducts induce p53 mutations—a very common characteristic found in hepatocellular carcinoma [43]. Yuan et al. identified a positive correlation between urinary 8-epi-prostaglandin F2α—a marker of lipid peroxidation—and the risk of developing liver cancer, regardless of smoking history, alcohol consumption, HBV infection, or liver cirrhosis [91].

It is known that high 8-OHdG serum levels represent a risk factor for the development of hepatocellular carcinoma in HCV-infected patients. In contrast, in hepatitis B, the role of 8-OHdG is unclear. Recently, it has been shown that HBx induces the accumulation of 8-OHdG in hepatocytes by inhibiting the activity of the enzymes MTH1 and MTH2 via hypermethylation. MTH1 and MTH2 are enzymes that play a crucial role in the DNA defense against oxidative stress. These enzymes prevent the incorporation of oxidized nucleotides into DNA. Interestingly, the increase in 8-OHdG levels caused by HBx is reversible; therefore, the upregulation of MTH1 and MTH2 expression leads to the normalization of 8-OHdG levels [92].

DNA hypomethylation has been shown to be one of the harmful effects of ROS on nucleic acids [93]. It seems that aberrant DNA methylation is an important process that contributes to the development of hepatocellular carcinoma [94]. It is well established that cyclin D1 plays an important role in carcinogenesis [95]. Hypomethylation of the cyclin D1 promoter takes place under oxidative stress conditions. Liu et al. emphasized that the plasma cyclin D1 methylation promoter could represent a more reliable diagnostic biomarker than the serum alpha fetoprotein [94]. They showed that this biomarker can differentiate patients with HBV-associated hepatocellular carcinoma from those with chronic hepatitis, as well as from healthy subjects [94].

## 7. Antioxidant Therapy in HBV Infection

It has been shown that antioxidant capacity in patients with hepatitis B is reduced. The serum levels of antioxidant compounds such as vitamin C, vitamin E, and glutathione were lower in patients with hepatitis B compared to healthy individuals [96]. Seen et al. suggest that antioxidant therapy can be used as an adjunctive therapy in patients with chronic hepatitis B [97]. The administration of antioxidant vitamins may have a protective effect against HBV-induced free radical liver injury [98]. Fiorino et al. have suggested that vitamin E modulates host microRNA synthesis at the post-transcriptional level, and that it may play an important role in the regulation of HBV replication in patients with persistent HBV infection [99]. Glutathione may inhibit viral replication. The interaction between heat shock protein-90 (HSP-90) and core protein is involved in HBV assembly. In the presence of glutathione, the conformation of HSP-90 is modified, which prevents the assembly of virions [98]. Qian et al. administered glutathione to a group of patients with chronic hepatitis B, and observed decreased levels of transaminases, bilirubin, pro-inflammatory interleukins IL-6 and IL- 8, TNF-α and TGF-β [100].

There is evidence that there are several other compounds with antioxidant properties that may be useful in the management of HBV infection. The administration of silymarin has been associated with decreased transaminase levels, and selenium appears to have a protective effect against liver cancer [66]. Extracts of leaves of *Moringa oleifera* exhibit antioxidant properties. The leaves of *M. oleifera* contain vitamins, carotenoids, polyphenols, phenolic acids, flavonoids, etc. Using Huh7 cells expressing HBV genotypes C or H, Feustel et al. have revealed that treatment with *M. oleifera* leaves may decrease fibrosis markers, IL-6, and HBsAg secretion [101].

## 8. Conclusions

Recent research emphasizes new markers of oxidative stress in HBV infection. Therefore, NOX-2, sE-cadherin, thiol–disulfide homeostasis parameters, glutamine, and nitrotyrosine are potential new markers for the evaluation of oxidative stress in patients with chronic HBV infection. The assessment of fibrosis by noninvasive tests could be performed using oxidative stress markers. In this regard, the researchers propose TAC, 8-oxo-Gsn and thiol–disulfide homeostasis parameters as promising markers for assessing the degree of fibrosis in patients with chronic hepatitis B. These data may be the basis for the development of new tools for the management of these patients.
